# Design and Fabrication of Vertically-Integrated CMOS Image Sensors

**DOI:** 10.3390/s110504512

**Published:** 2011-04-27

**Authors:** Orit Skorka, Dileepan Joseph

**Affiliations:** Department of Electrical and Computer Engineering, University of Alberta, Edmonton, AB T6G 2V4, Canada

**Keywords:** CMOS image sensors, photodetectors, logarithmic sensors, stacked ICs, flip-chip bonding

## Abstract

Technologies to fabricate integrated circuits (IC) with 3D structures are an emerging trend in IC design. They are based on vertical stacking of active components to form heterogeneous microsystems. Electronic image sensors will benefit from these technologies because they allow increased pixel-level data processing and device optimization. This paper covers general principles in the design of vertically-integrated (VI) CMOS image sensors that are fabricated by flip-chip bonding. These sensors are composed of a CMOS die and a photodetector die. As a specific example, the paper presents a VI-CMOS image sensor that was designed at the University of Alberta, and fabricated with the help of CMC Microsystems and Micralyne Inc. To realize prototypes, CMOS dies with logarithmic active pixels were prepared in a commercial process, and photodetector dies with metal-semiconductor-metal devices were prepared in a custom process using hydrogenated amorphous silicon. The paper also describes a digital camera that was developed to test the prototype. In this camera, scenes captured by the image sensor are read using an FPGA board, and sent in real time to a PC over USB for data processing and display. Experimental results show that the VI-CMOS prototype has a higher dynamic range and a lower dark limit than conventional electronic image sensors.

## 1. Introduction

Fabrication of integrated circuit (IC) devices in 3D structures, where active components are stacked vertically to form a microsystem, is a growing trend in IC design. The approach offers several advantages over planar technologies. First, only one package is required for several dies, which makes it possible to build lighter and more compact systems [1]. Second, because the long traces on a printed circuit board (PCB) are replaced by much shorter connections between dies, the resistance (R) and capacitance (C) of interconnects are significantly lowered. This results in a notable reduction in transmission power loss. Moreover, RC delays become smaller and, therefore, the interconnect bandwidth increases [2]. In addition, the information flow between dies may be raised substantially with vertical integration because the number of connections between dies is area-limited, not perimeter-limited as with planar technologies.

Image sensors are likely to benefit from the vertical integration because each tier can be fabricated in a technology optimized for the type of devices it contains. Image sensors require photodetectors for sensing, analog circuits for amplification and pre-processing, and digital circuits for control and post-processing. While digital circuits may exploit the advantages of a nanoscale CMOS process, photodetectors may be fabricated in a larger scale process. Analog circuits may be fabricated in an intermediate scale process or, with robust design methods, in the same process as the digital ones. Furthermore, in some fabrication methods, the photodetector tier need not use crystalline silicon, which makes it easier to target invisible bands of the electromagnetic spectrum.

For vision applications, image sensors should have features such as high spatial and temporal resolution, high signal-to-noise ratio, high dynamic range, and low dark limit. Advanced pixel-level circuitry, such as digital pixel sensors (DPS), may be used to address these competing requirements. With CCD technology, however, standard CMOS circuits may not be integrated either in the pixel or elsewhere on the chip. Although DPS is possible with CMOS technology, in-pixel circuits and photodetectors must be laterally integrated. Thus, it is impossible to use advanced circuitry without either having impractical pixel dimensions or using a nanoscale CMOS process, which is less suitable for photodetection. Scaling down the CMOS process involves shallower diffusion layers and increased levels of channel doping. This results in increased dark noise, which degrades photodetection in the dark [3].

The idea to build electronic image sensors by vertical stacking of active components is not new. There are published works from as early as the late 1970s that describe vertically-integrated (VI) image sensors for the infrared (IR) band, where readout is done using silicon CCDs. These VI-CCD image sensors were made either by direct deposition of IR photodetectors on silicon CCDs or by bonding a substrate with IR photodetectors to a substrate with CCDs using solder bumps [4]. Following the emergence of the CMOS APS technology, CMOS readout circuits increasingly were used in VI image sensors that targeted invisible bands. For example, Bajaj describes VI-CMOS image sensors for the IR band in 2000 [5].

Although the motivation for VI-CMOS image sensors started with imaging in invisible bands, where the optoelectronic properties of crystalline silicon make it unsuitable for photodetection, the advantages offered by vertical integration have attracted international research groups since the late 1990s to use this approach also for imaging in the visible band. Examples include: (1) the work done by Benthien *et al.* [6], who used the direct deposition method, which they named “thin film on ASIC” (TFA); (2) image sensors presented by Rockwell Scientific (now a part of Teledyne) [7] that were fabricated using the solder bump or flip-chip bonding method; and (3) the image sensor shown by Lincoln Laboratories in MIT [8] that was based on the through-substrate-via (TSV) approach.

Canadian research institutions have also developed and demonstrated devices that are based on vertical integration of a sensor array and a readout circuit array. Aziz *et al.* [9] present a 3D microsystem for multi-site extra-cellular neural recording that is composed of a CMOS die and an electrode die, where the two are flip-chip bonded using gold stud-bumps. Izadi *et al.* [10] present an image sensor for medical X-ray imaging. It is composed of amorphous-selenium photodetectors deposited on amorphous-silicon thin film transistors (TFT) that are used for readout. INO, a Quebec firm, has integrated uncooled bolometric sensors, which are micro-electro-mechanical systems (MEMS), and CMOS readout circuits. These VI-CMOS image sensors are designated for the IR and THz bands [11,12].

**Figure 1.**
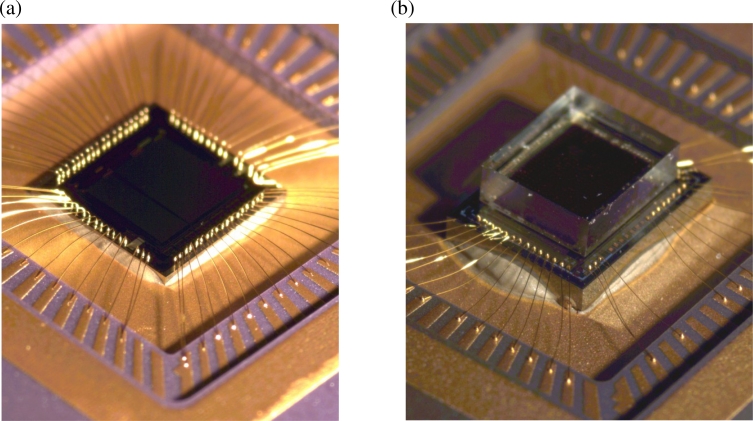
**(a)** CMOS and **(b)** VI-CMOS image sensors designed at the University of Alberta and fabricated via CMC Microsystems.

Figure 1 shows a VI-CMOS image sensor, made by flip-chip bonding, next to a CMOS image sensor. Both were designed at the University of Alberta (UofA) and fabricated via the Canadian Microelectronics Corporation (CMC). The CMOS sensor was fabricated in a 0.35 *μ*m TSMC process. Each pixel contains a photodetector integrated laterally with CMOS transistors. The VI-CMOS sensor comprises a CMOS die (bottom) and a photodetector die (top) that were assembled by flip-chip bonding. Whereas the CMOS die was fabricated in a standard 0.8 *μ*m Dalsa process, the photodetector die was fabricated in a custom process via Micralyne Inc. and the UofA Nanofab. Each pixel contains a photodetector integrated vertically with CMOS transistors.

This paper is an extended version of a CMC application note [13]. The work describes the first process flow for VI-CMOS image sensors made through CMC, an umbrella organization for Canadian Microsystems. Unlike the note, this paper provides experimental results for a prototype realized using the process flow. Section 2 discusses optional fabrication methods for VI-CMOS image sensors and focuses on general principles in the design of those made by flip-chip bonding. It includes a detailed review on various choices for the photodetector die. Section 3 presents, as a specific example, the design and fabrication of the VI-CMOS image sensor prototype shown in Figure 1(b). Finally, Section 4 describes the digital camera that was developed to test the prototype, and presents results obtained from the VI-CMOS image sensor characterization.

## 2. Principles of Design and Fabrication

**Figure 2.**
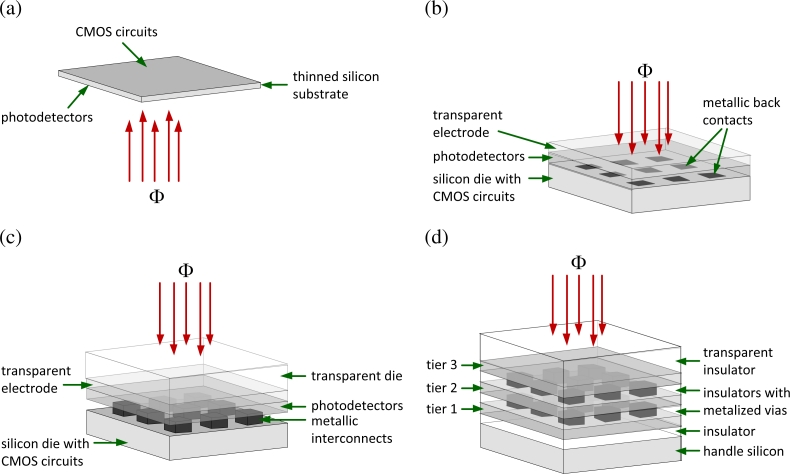
VI-CMOS image sensors fabricated by **(a)** thinned substrate, **(b)** thin-film-on-ASIC (TFA), **(c)** flip-chip, and **(d)** through-substrate via (TSV) technologies.

VI-CMOS image sensors may be designed for different fabrication methods, as shown in Figure 2. With thinned substrate technology [14], after CMOS circuits are formed on one side, photodetectors are formed on the other side of a silicon substrate that is also thinned. With TFA technology, thin films that define photodetectors are deposited and patterned directly on a silicon substrate with CMOS circuits, *i.e.*, an ASIC. In these two technologies, semiconductor devices are vertically integrated on one die, enabling monolithic VI-CMOS image sensors.

With flip-chip technology, VI-CMOS image sensors are composed of two dies: a silicon die with CMOS circuits and a transparent die with photodetectors. After separate fabrication, the two are precisely aligned and attached face-to-face using metallic interconnects. TSV technologies may be used to vertically integrate two or more dies. Front-to-back electrical connections between dies are possible by etching holes through substrates and metalizing them. Burns *et al.* [8] demonstrated a TSV image sensor with three tiers using stacked silicon-on-insulator (SOI) technology. Top, middle, and bottom tiers were dedicated to photodetectors, analog circuits, and digital circuits, respectively. While all tiers were fabricated using SOI substrates, each tier had its own process scale.

Our first efforts toward a prototype concerned TFA technology. However, TFA involves extensive post-processing of finished CMOS substrates, including surface planarization and film deposition. Process development requires whole CMOS wafers but we could obtain only tens of CMOS dies at a relatively low cost using a multi-project wafer service through CMC. Furthermore, because the CMOS dies were fabricated in a commercial process, the exact materials and dimensions used were trade secrets, which made it more difficult to develop compatible post-processing.

In 2007, we switched to flip-chip technology because it was the only way to make a VI-CMOS image sensor with the support of CMC. At the time, TSV technologies were still in development—there were no TSV services available through CMC. Flip-chip technology required the design and fabrication of a CMOS die (Section 2.1) and a photodetector die (Section 2.2), which are then bonded (Section 2.3) to assemble a VI-CMOS image sensor.

### 2.1. CMOS Die

**Figure 3.**
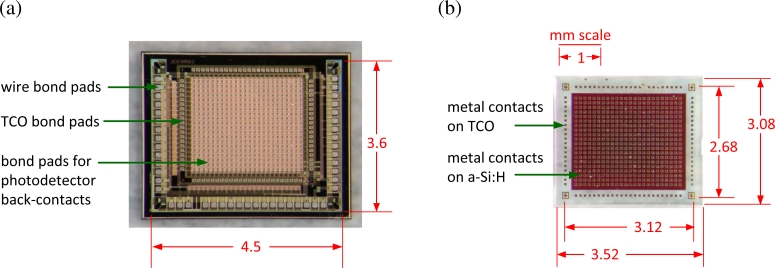
The VI-CMOS prototype in Figure 1(b) is composed of **(a)** a CMOS die and **(b)** a photodetector die.

The CMOS die in Figure 3(a) was designed for a standard CMOS process. Its central area contains a circuit array for readout purposes, which mates to back contacts on the photodetectors. Surrounding bond pads mate to a transparent conductive oxide (TCO), which defines a front contact on the photodetectors. Peripheral bond pads are required to wire the image sensor to a package. Design of a CMOS die for a VI-CMOS image sensor is similar to the design of a CMOS image sensor, but there are some important differences.

Typical CMOS image sensors are composed of: active pixels, which amplify photodetector signals; row and column address decoders, which select pixel signals for readout; column and output buffers, which route selected signals to output buses; and analog-to-digital converters (ADC), which transform the output signals. In addition to the photodetectors, these readout circuits define how photogenerated charge carriers are interpreted. In general, the digital response is a linear or logarithmic function of the light stimulus. Usually, a few ADCs are included for all pixels. However, designs that include one or two ADCs per column, or column-level ADCs, are increasingly common. Further details on CMOS image sensor design may be found in the literature [15–17].

As with a CMOS image sensor, the floor plan of a CMOS die designed for a VI-CMOS image sensor also requires an active pixel array, address decoders, buffers, and one or more ADCs. However, unlike CMOS image sensors, there is no photodetector in the pixel layout. Instead, each pixel has a bond pad to form an electrical contact with a vertically-integrated photodetector after flip-chip bonding. This makes a bond-pad array of photodetector back-contacts. Surrounding bond pads mate to a transparent conductive oxide (TCO), which defines a front contact on the photodetectors. Like typical CMOS chips, a VI-CMOS image sensor also requires peripheral bond pads for wire bonding to a package that can be soldered onto a PCB.

For visible-band image sensors, the motivation for VI-CMOS over CMOS technology is to facilitate one ADC per pixel. With conventional CMOS image sensors, analog signals must travel outside the pixel array for conversion to digital signals. While traveling, they accumulate noise. Because digital signals are far more immune to noise than analog ones, the signal-to-noise ratio is expected to improve with pixel-level ADCs. However, as ADCs require complex circuits, building them in a CMOS technology suitable for visible-band imaging implies a relatively low spatial resolution. Further details on pixel-level ADCs may be found in the literature [18,19].

In CMOS image sensors, borders of each photodetector are defined in the pixel layout. In VI-CMOS image sensors, however, there are good reasons to avoid physical borders between adjacent photodetectors. The manufacturing cost of the photodetector die may be reduced by avoiding the lithography steps required to pattern the borders. Moreover, edges of patterned devices introduce defect states and other imperfections that degrade performance, for example, by increasing the dark currents of photodetectors.

Without physical borders between adjacent photodetectors, lateral currents may flow due to drift and diffusion. This would cause photogenerated charge carriers to enter the “wrong” pixels of the CMOS die, a condition known as “crosstalk”. The crosstalk may be made negligible if a vertical electric field of sufficient uniformity and magnitude is applied on all photodetectors by the CMOS circuits. Schneider *et al.* [20] used a feedback active pixel to introduce this approach in a VI-CMOS image sensor made by TFA technology. Skorka and Joseph [21] elaborated on the design of such pixels, especially in terms of stability and compensation.

### 2.2. Photodetector Die

The photodetector die in Figure 3(b) was fabricated in a custom process. Its central area has an array of bond pads on a light-sensitive semiconductor; the surrounding bond pads are on the TCO. Unlike with the CMOS die, the challenge with designing this die has to do with its cross-section, and not its floor plan. One must specify the material layers, their ordering, and their thicknesses. Usually, the electric field in the photodetectors is oriented parallel to the incident light flux, *i.e.*, parallel to Φ in Figure 2(c). Otherwise, each pixel requires two bond pads.

#### 2.2.1. Handle Substrate

The handle substrate of the photodetector die must be transparent for the electromagnetic band targeted by the application. For better imaging performance, a large percentage of photons must reach the light-sensitive devices. There is always some loss of photons due to reflections at interfaces formed in the path of the light. However, loss of photons due to absorption in the handle substrate should be minimized.

Handle substrates of the photodetector and CMOS dies should have similar coefficients of thermal expansion (CTEs). Large CTE differences cause mechanical stress when the temperature of the assembled device varies from the temperature of assembly. Temperature changes are also expected when the device is powered up or down. Mechanical stress results in distortion of features, which may affect functionality, especially with nanoscale CMOS. Table 1 gives three CTEs of silicon, which is the handle substrate of standard CMOS dies, and of substrates suitable for visible-band applications. Borosilicate glass, which is sold commercially under brand names such as Pyrex and Borofloat, has CTEs closest to those of silicon.

When selecting a handle substrate, properties of other substrate materials in the photodetector die should be considered. Amorphous materials may, in general, be deposited on any handle substrate. However, crystalline materials require handle substrates with matching lattice constants. Moreover, the handle substrate must withstand all process steps required to make the photodetector die. For example, polysilicon films, which are suitable for photodetection, may be deposited using low-pressure chemical vapour deposition (LPCVD) at over 600 ^°^C. If the films are doped, they require annealing at 900–1,000 ^°^C for dopant activation. Borosilicate glass, although transparent and with CTEs close to those of silicon, cannot be used with polysilicon photodetectors because it cannot withstand these temperatures. Fused silica, quartz, or sapphire should be used in this case.

#### 2.2.2. Transparent Electrode

The first layer on the handle substrate must be a transparent conductor. It forms the front contact of all photodetectors, and is an essential electrode to realize a vertical electric field. In some cases, it is possible to use a heavily-doped section of the handle substrate or the light-sensitive devices (subsequent layers) for this purpose. In other cases, one deposits a film based on thin metals, transparent conductive oxides (TCOs), transparent conductive polymers (TCPs), or carbon nanotubes (CNTs). These materials are described below.

**Thin metals**: Metals are very good conductors but are opaque to visible light. Metal films, however, transmit some visible light if they are very thin. Aluminum (Al), silver (Ag), and gold (Au) are attractive choices because they have a relatively high transmittance in the visible band. These metals must be less than 20 nm thick to have at least 10% transmission [23,24]. Unfortunately, thin films are much less conductive than thick ones, and their conductivity is much more sensitive to thickness variation. Hence, it may be difficult to achieve a satisfactory combination of transparency, conductivity, and uniformity with thin metals [25].

**Transparent conductive oxides**: TCOs are semiconductors, usually polycrystalline or amorphous, that have high optical transparency and high electrical conductivity, properties normally considered mutually exclusive [26]. To be used as a TCO, a semiconductor needs a high band gap (≳ 3.1 eV), a high concentration of free carriers (≳ 10^19^ cm^−3^)—*i.e.*, it needs to be a degenerated semiconductor [27]—and a good mobility (≳ 1 cm^2^V^−1^s^−1^). Popular TCOs are indium oxide (In_2_O_3_), tin oxide (SnO_2_), and zinc oxide (ZnO), which are all n-type semiconductors. Table 2 presents their optoelectronic properties. Although TCOs are more conductive than typical semiconductors, they are much less conductive than metals.

Often, TCOs are doped with impurities. Widely used examples are tin-doped indium oxide (In_2_O_3_:Sn or ITO) and aluminum-doped zinc oxide (ZnO:Al or AZO). ITO has been used for many years in applications where transparent electrodes were needed. However, because indium and tin are expensive metals, while zinc is cheap and non-toxic, AZO films have been getting more attention in recent years [28].

**Transparent conductive polymers**: Organic electronic devices are based on polymers such as those listed in Table 3. Mass production of organic devices is expected to be cheaper than that of inorganic devices. Moreover, polymers are ideal for realizing flexible devices. Currently, ITO is widely used as a transparent electrode in organic optoelectronic devices [29]. However, ITO is brittle, which makes it unsuitable for flexible devices. PEDOT:PSS is a flexible TCP that has been touted as a suitable replacement [30].

At present, the conductivity of TCPs is about an order of magnitude lower than that of ITO [31], and TCPs are less transparent to visible light than ITO [30]. Moreover, when a device includes polymers, the maximum temperature that it can withstand during fabrication and operation is more limited. Therefore, the advantages of working with TCPs are relevant mainly when the whole device is organic.

**Carbon nanotubes**: Researchers have shown recently that thin films of CNTs, mainly single-walled CNTs (SWCNTs), may be used as transparent electrodes [32,33]. SWCNTs are attractive because they can be deposited on almost any substrate [34], and because their mechanical properties make them suitable for use in flexible devices. Whereas indium prices are rising due to the increasing depletion of indium sources worldwide, carbon remains an abundant element. Hence, SWCNTs have a promising future.

Similar to the difficulties faced with polymers, the transparency and conductivity of SWCNTs are inferior to those of ITO. Sangeeth *et al.* [30] compared experimentally the performance of ITO, PEDOT:PSS, and SWCNTs. When SWCNT films have a transparency comparable to that of ITO films, for light at 550 nm (*i.e.*, the middle of the visible band), their conductivity is almost two orders of magnitude lower. Nonetheless, researchers are working on methods to improve the conductivity of CNT films [34,35].

#### 2.2.3. Light-Sensitive Devices

Electronic photodetectors are mainly constructed from a light-sensitive semiconductor, which must have high absorption coefficients for the targeted wavelengths. Hence, for visible-band imaging, the semiconductor band gap must be smaller than the energy of red photons. In addition, absorbed photons must change the electrical properties of the semiconductor sufficiently so that the change is detectable by a CMOS circuit.

With VI-CMOS image sensors, there may be more degrees of freedom in photodetector design than with CMOS image sensors. For example, the depth of lateral photodetectors in a CMOS image sensor is largely fixed by the doping profiles of the CMOS process. However, the depth of vertical photodetectors in a flip-chip image sensor is largely variable. On the photodetector die, the thickness of the light-sensitive semiconductor may be chosen to optimize a performance measure, such as the ratio between photocurrent and dark current [36].

In addition to layer thicknesses, a photodetector design must specify the device type and the layer materials. In general, light-sensitive devices may be categorized as photoconductors, photodiodes, or phototransistors [37]. Traditionally, photodetector layers were based on inorganic semiconductors, either crystalline or amorphous ones, but organic semiconductors may also be used. Further details are given below.

**Photoconductors**: A photoconductor (or photoresistor) consists of a uniformly-doped semiconductor sandwiched between ohmic contacts. Device conductivity increases with increasing illumination. With an applied electric field, photogenerated electrons and holes are collected by opposite contacts. For good performance, the charge carriers should have long lifetimes and high mobilities. Otherwise, most of the excess electron-hole pairs recombine on their way to the contacts, and do not contribute to the photocurrent. The semiconductor should have a low noise-current in the dark, with respect to photocurrent, for the device to have an acceptable response in dim illumination.

**Photodiodes**: Photodiodes are commonly used in CMOS image sensors. They incorporate either p-n junctions between p-doped and n-doped semiconductors or Schottky junctions between semiconductors and metals. Under reverse bias, photodiodes usually have lower dark currents than comparable photoconductors because of a depletion layer. An electric field accelerates photogenerated charge carriers toward the contacts, where they contribute to photocurrent. To increase the thickness of the depletion layer, an intrinsic layer (undoped or lightly doped) may be inserted between the p and n regions. This makes a p-i-n photodiode.

Avalanche photodiodes permit the detection of single photons. These devices realize high gains by accelerating photogenerated charge carriers, using a high electric field, so as to generate secondary electron-hole pairs in the depletion layer. Lately, there has been an increased interest in avalanche photodiodes [38], which have applications also in lens-less imaging systems, e.g., in microfluidic devices (lab on a chip).

**Phototransistors**: The term phototransistor is normally used for two back-to-back p-n junctions, *i.e.*, light-sensitive devices that resemble bipolar transistors. When two Schottky junctions are used, the device is often called a metal-semiconductor-metal (MSM) photodetector. In either case, one junction is reverse biased while the other is forward biased when a voltage is applied. The floating-base configuration is often used.

**Crystalline semiconductors**: Crystalline silicon is the material used to make photodetectors in standard CMOS and CCD image sensors. Other crystalline semiconductors that are suitable for photodetection in the visible band are alloys like gallium arsenide [39] (GaAs) and indium gallium nitride [40] (InGaN). Common deposition methods for these materials are molecular beam epitaxy (MBE) and metal-organic chemical vapour deposition (MOCVD). Mercury cadmium telluride (HgCdTe or MCT) has long been used for infrared photodetection. The band gap of this alloy may be varied by changing the element proportions [41]. The main drawback with crystalline materials is that they can be deposited only on substrates with similar lattice constants. Moreover, the deposition needs to be done at relatively high temperatures.

**Amorphous semiconductors**: Hydrogenated amorphous silicon (a-Si:H) is an amorphous semiconductor commonly used for photodetection in the visible band. It is a relatively cheap material, has a high absorption coefficient for visible light, and can be deposited on various substrates. Popular deposition methods for a-Si:H optoelectronic devices are sputtering and plasma-enhanced chemical vapour deposition (PECVD). The deposition is done at relatively low temperatures, *i.e.*, at 200–250 ^°^C. Amorphous selenium (a-Se) is another amorphous semiconductor that is used for photodetection. Its properties make it ideal for detecting X-rays [42]. However, a-Se photodetectors for the visible band have also been demonstrated [43].

**Organic semiconductors**: Although organic semiconductors have been studied for 60 years, their use in optoelectronic devices, e.g., LCD displays, is quite recent. The breakthrough was the discovery that some organic semiconductors are photoconductive under visible light [44]. Initially, organic semiconductors were unstable and had a low carrier mobility. However, their properties have improved in recent years thanks to extensive research. They are attractive for use in optoelectronic devices, as an alternative to inorganic semiconductors, because of their low cost, low deposition temperature, and flexibility. Organic photodetectors for the visible band have been demonstrated using materials such as pentacene [45], a blend of PDDTT and PC60BM [46], and a structure composed of P3HT and CuPc thin films [47]. Deposition methods for organic semiconductors include thermal evaporation, organic molecular beam deposition (OMBD), and spin coating. In some cases, deposition may be done at or just above room temperature.

### 2.3. Flip-Chip Bonding

**Figure 4.**
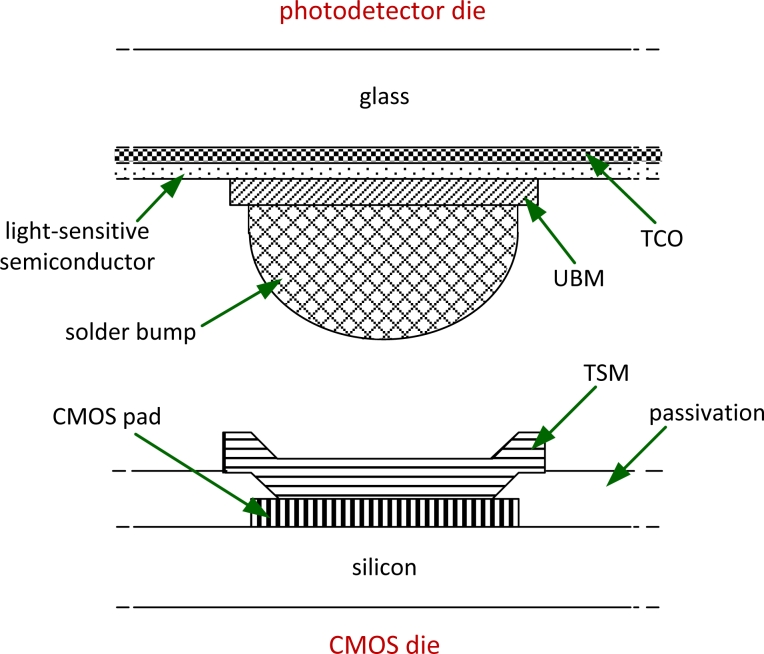
Pre-processing required for flip-chip bonding (not drawn to scale). TSM is deposited on the CMOS die, and UBM is deposited on the photodetector die. Solder bumps, tens of microns thick, are fabricated on the UBM.

CMOS dies and photodetector dies need to undergo a few more process steps before they can be flip-chip bonded. The process performed on the CMOS dies includes etching of the native oxide layer from the aluminum bond pads and deposition of a metal stack, called top surface metallurgy (TSM), that has a good wettability to the solder material used in the flip-chip bonding. Photodetector dies are processed to form two sets of bond pads, which are also metal stacks, called under bump metallization (UBM). These bond pads form back contacts on the photodetectors, and also connect to the transparent electrode, which makes the front contact of the photodetectors. The UBM must have a good adhesion to non-metallic materials, such as semiconductors and conductive oxides, as well as good wettability to the solder material. Solder bumps are then formed on the UBM, as illustrated in Figure 4. Having the solder bumps on the smaller die facilitates the assembly process.

## 3. Design and Fabrication of a Prototype

The previous section focused on general principles in the design and fabrication of a VI-CMOS image sensor. This section focuses on the design and fabrication of a specific prototype.

CMOS dies were fabricated in a standard CMOS process. Therefore, the challenging part with these dies was the circuit design, mainly the pixel layout, and not the fabrication. Photodetector dies, however, were fabricated in a custom process. In terms of manufacturability and performance, these dies are not the best that could be designed for the visible band (400–700 nm), which was targeted for simplicity. However, they are the best that could be made with the available materials and equipment. A new process was developed at the UofA Nanofab to realize the photodetector dies. Process development requires that all materials used, e.g., etching gases and solutions, and all conditions reached, e.g., maximum temperature, work without any undesirable side effects.

### 3.1. CMOS Die

The CMOS die was designed for a 0.8 *μ*m Dalsa process, which has three metal layers. In this process, CMOS devices are fabricated in a large N well. Therefore, NMOS transistors require a P well, whereas PMOS transistors are fabricated in the substrate. The supply voltage *V*_dd_ is 5 V.

**Figure 5.**
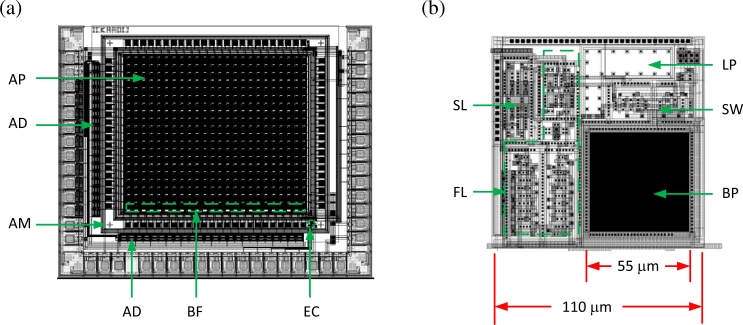
**(a)** Floor plan and **(b)** pixel layout of the designed CMOS die.

A floor plan of the design is shown in Figure 5(a). It includes a 20 × 24 array of active pixels (AP), row and column address decoders (AD), buffers (BF), extra circuits (EC) for test purposes, and alignment marks (AM). ADCs were not included for simplicity. Schematic and layout designs were done with Cadence. The schematic was verified using DC, AC, and transient simulations. The layout was verified using design rule check (DRC) and layout versus schematic (LVS) tests. Dies were fabricated through CMC.

**Figure 6.**
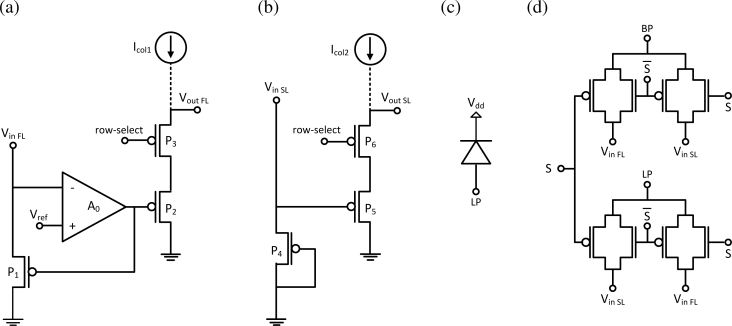
Principle schematics of the designed CMOS die: **(a)** the feedback logarithmic-response circuit (FL); **(b)** the standard logarithmic-response circuit (SL); **(c)** the lateral photodiode (LP); and **(d)** the switch (SW).

Layout of the active pixel is shown in Figure 5(b), and the principle schematics of each block is shown in Figure 6. Each pixel has a bond pad (BP) for integration with a vertical photodetector and a lateral photodiode (LP). It also includes a feedback logarithmic-response circuit (FL), a standard logarithmic-response circuit (SL), and a switch (SW) that configures the output. Although electrostatic discharge protection is recommended for all bond pads, such circuits were only included in wire bond pads. Interior bond pads are inaccessible after flip-chip bonding.

Because the light-sensitive semiconductor in the photodetector die is unpatterned, active pixels in the CMOS die employ feedback circuits to reduce crosstalk. A logarithmic response to light stimulus was chosen over a linear one because it can capture a higher dynamic range. The feedback logarithmic-response circuit maintains a constant voltage at the photodetector back contacts and, therefore, uses current as its input signal.

Readout of the FL and SL circuits is activated when the *row*-*select* signal is logic low. In this case, transistors *P*_3_ and *P*_6_ are conducting, and column bias currents, *I*_col1_ and *I*_col2_, flow through the source-follower transistors, *P*_2_ and *P*_5_, respectively. Each pixel has two output lines, where one is coming from the FL circuit, *V*_out FL_, and the other is coming from the SL circuit, *V*_out SL._

A lateral photodiode and a standard logarithmic-response circuit are included in each pixel so that the functionality of the CMOS die could be tested independently of flip-chip bonding and feedback. The switch in each pixel is configured externally through the control line *S*. In one configuration, the lateral photodiode is connected to the input node of the standard logarithmic circuit, *V*_in SL_, and the vertical photodetector is connected to the input node of the feedback logarithmic circuit, *V*_in FL_. Connections are swapped in the second configuration. The switch acts as a multiplexer to analog signals; it is composed of transmission gates.

Pixels are 110 × 110 *μ*m^2^, which is quite large for visible-band applications. When the project was at the design stage, CMC could guarantee flip-chip bonding only for bond pads of at least 55 *μ*m pitch and 110 *μ*m spacing from centre to centre. However, pixel dimension of 10 × 10 *μ*m^2^, *i.e.*, small enough for imaging in the visible band, have been demonstrated with VI-CMOS image sensors made by flip-chip bonding [48].

In general, design rules of CMOS processes do not allow placement of devices underneath bond pads, and require bond pads to connect to all metal layers. However, researchers are working to change this. For example, Ker *et al.* [49] designed and tested NMOS transistors underneath wire bond pads. Their bond pads used all metal layers except the lowest, which was used for the transistors. Even after wire bonding, there was little difference between the characteristics of these transistors and standard ones, located far from the bond pads.

### 3.2. Photodetector Die

The design of the photodetector die was mainly determined by the light-sensitive semiconductor that we could use. There was no equipment for GaAs deposition in the Nanofab. Moreover, GaAs films must be deposited on GaAs substrates, which are opaque to visible light. Some options, such as HgCdTe, were ruled out because of their toxicity. Other options, such as organic films, did not have good enough performance at the time. After a careful review, the only semiconductor we could work with productively was a-Si:H.

In general, a-Si:H can be deposited either by sputtering or by PECVD. The latter method tends to yield higher quality films than the former method. Sputtering must be done at 200–250 ^°^C as a reactive process using hydrogen. Although the Nanofab has sputtering machines, none of them had a hydrogen supply. Fortunately, Micralyne Inc., an Edmonton company, agreed to deposit a-Si:H films with their PECVD machine. Micralyne’s process, however, did not support dopant gases. Therefore, our devices had to be based on intrinsic films, and so p-n or p-i-n photodiodes could not be implemented. Consequently, we designed an MSM device, in which an intrinsic a-Si:H layer is sandwiched between two conductive layers.

**Figure 7.**
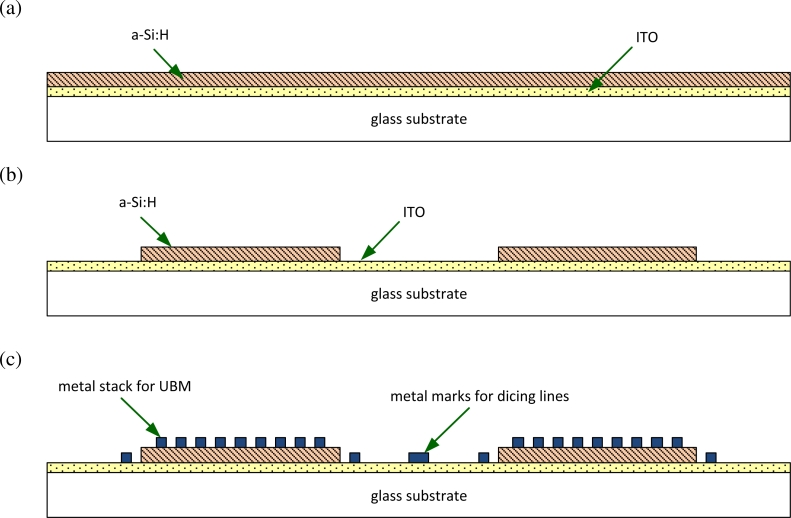
Fabrication process of the designed photodetector die: **(a)** deposition of ITO and a-Si:H; **(b)** patterning of the a-Si:H; and **(c)** deposition and patterning of metal.

Figure 7 illustrates the fabrication process of the photodetectors. ITO and a-Si:H were deposited on the handle substrate by sputtering and PECVD, respectively. The purpose of the first lithography step was to selectively etch the a-Si:H layer. One needs to expose the ITO layer because, in the VI-CMOS image sensor, an electric potential must be applied to it. The a-Si:H was dry etched using the Plasma Lab *μ*Etch machine in the Nanofab. The chamber was pumped down prior to the process. Etching was done in an atmosphere composed of 40 sccm of carbon tetrafluoride (CF_4_) and 10 sccm of oxygen (O_2_). The CF_4_/O_2_ plasma also serves as surface treatment to improve performance of the ITO film [50]. An RF power of 100 W was applied, and the chamber pressure was 63 mTorr. A chrome mask was used for the dry etch because earlier trials with a photoresist mask showed that the etchant gases consumed the photoresist at a higher rate than the a-Si:H.

The final photodetector design was a Cr/a-Si:H/ITO stack on glass. Chrome was used as the back contact because it has a good adhesion to non-metal substrates, including a-Si:H. To get a higher photocurrent to dark current ratio with this MSM device, the CMOS die connects the ITO electrode to a higher voltage than the chrome electrode due to the relative size of potential barriers at the two Schottky junctions [51].

#### 3.2.1. Handle Substrate

**Figure 8.**
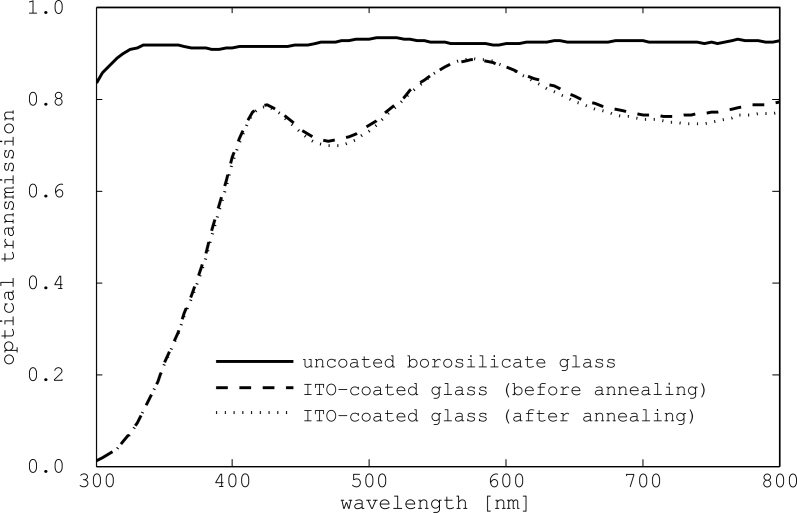
Optical transmission of a borosilicate glass substrate before and after ITO deposition. Transmission of the coated glass was measured before and after the ITO was annealed.

We used borosilicate glass (Borofloat) as the handle substrate for the photodetectors. Although thinner substrates were available, we used 1 mm thick ones because they were in stock at the Nanofab. Substrates were cleaned using a Piranha solution (sulfuric acid and hydrogen peroxide). Using the Woollam VASE ellipsometer in the Nanofab, we measured the optical transmission of a naked substrate. Results are presented in Figure 8. They show that 90% of visible light is transmitted.

#### 3.2.2. Transparent Electrode

Equipment and materials available in the Nanofab meant we could use either a thin metal or TCO film as the transparent electrode. The layer could be realized by physical vapour deposition (PVD), *i.e.*, either sputtering or e-beam evaporation. We preferred the TCO option because our first sputtering trials of ITO were successful, despite a brittle ITO target. Moreover, if a metal film is used, it must be less than 20 nm thick. Although the substrate is rotated during the deposition, there are still non-uniformities in film thickness. With thin metals, small variations in thickness result in large variations in transparency and conductivity.

For photodetectors based on a-Si:H, in which an a-Si:H film is deposited on a TCO substrate, ZnO is preferable to ITO as the TCO material. The a-Si:H is normally deposited using a PECVD process, during which the TCO surface is exposed to hydrogen plasma. When ITO is exposed to hydrogen plasma, hydrogen radicals react with the oxygen in the ITO, and reduce some of the oxide into metals, *i.e.*, indium and tin [52,53]. This decreases the transparency of the ITO to visible light, and also changes the electrical properties of the a-Si:H/ITO contact. ZnO, on the contrary, is non-reactive under these conditions [54].

Although ZnO (and AZO) targets are available commercially, we were not allowed to work with zinc in the multi-user machines of the Nanofab because zinc has a high vapour pressure at low temperatures. Usage of zinc in the vacuum chambers would mean that, for a long time, future users of the machine would have zinc contamination in their depositions. Therefore, we had to work with ITO.

The ITO films were deposited in a Lesker magnetron sputtering machine with a Lesker ITO target. Prior to deposition, the chamber was pumped down to a pressure of 2 *μ*Torr. The deposition was done at room temperature in a pure argon environment with a gas flow of 50 sccm, and under pressure of 5.3 mTorr. Each deposition lasted for 50 min. An RF power of 80 W was used during the process. Under these conditions, the mean deposition rate of the ITO was 5.5 nm/min. Film resistivity was measured immediately after deposition using a four-point probe. The average value was 5.83 × 10^−4^ Ω cm. Deposition of ITO films in a reactive process, where the chamber atmosphere was 1% oxygen, resulted in films that were about twice as resistive (or half as conductive).

After deposition, the ITO films were annealed for two hours in air at 150–175 ^°^C. The average resistivity after annealing was 5.45 × 10^−4^ Ωcm. Annealing trials that were performed at 250–325 ^°^C instead resulted in increased resistivity. As shown in Figure 8, annealing had negligible impact on film transparency. ITO films show high optical transmission for wavelengths longer than 400 nm, and this makes them suitable for photodetection in the visible and near IR bands.

#### 3.2.3. Light-Sensitive Devices

Micralyne deposited two sets of a-Si:H films for us by PECVD. Both depositions were done at 200 ^°^C. In the first set, a-Si:H was deposited on two thermal-oxide silicon wafers. One film was 50 nm thick, and the other was 1, 000 nm thick. The purpose was to characterize the films, and to determine their suitability for the VI-CMOS image sensor prototype. In the second set, a-Si:H was deposited on four ITO-coated Borofloat wafers. Film thicknesses were 250, 500, 750, and 1, 000 nm. These depositions were used to fabricate the photodetector dies. The 1, 000 nm film in this second set, however, was not uniform over the substrate. “Bald” areas could be seen. We asked for multiple thicknesses to experimentally determine the optimal photodetector thickness.

**Figure 9.**
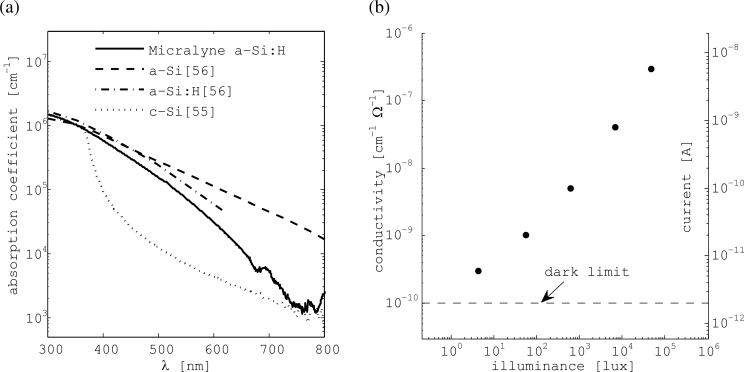
Optoelectronic properties of Micralyne a-Si:H films: **(a)** measured absorption coefficient as compared to literature values; and **(b)** film conductivity and estimated pixel current for varying surface illuminance.

The thin Micralyne film on the thermal oxide substrate was used to measure optical properties in the visible band. The absorption coefficient, *α*, could be extracted using the Woollam VASE ellipsometer and its accompanying software. A thin film is needed to ensure that not all the light passing through the a-Si:H is absorbed. Monochromatic light reflected from the sample at various interfaces contains information that is used to extract *α*. Figure 9(a) gives the absorption coefficient of the Micralyne film versus wavelength, *λ*. Results are compared to reported values for crystalline silicon [55], as well as hydrogenated and non-hydrogenated amorphous silicon [56]. In most of the visible band, the Micralyne film absorbs about ten times as much light as does crystalline silicon.

The thick Micralyne film on the second thermal oxide substrate was used for optoelectronic characterization. Because the thermal oxide substrate is an insulator, electrical properties of the film could only be tested with surface contacts. The transmission line model (TLM) method [57] was used to extract sheet resistance. This method requires long contacts with variable spacing to be patterned. Aluminum was deposited on the a-Si:H to form the contacts, and this was followed by a single lithography step. Aluminum interacts with a-Si:H to form ohmic contacts even at low temperatures [58]. Given film thickness, the material conductivity may also be extracted.

Measurements with the patterned Micralyne film were repeated for several levels of surface illuminance. The light source included a halogen light bulb with a 3,050 K correlated colour temperature and a cold fiber waveguide. Electrical measurements were performed using a probe station and a HP 4156 parameter analyzer. To estimate surface illuminance, luminance was measured with a meter from light reflected off white paper that was illuminated in identical conditions to the sample. Results are shown in Figure 9(b).

Conductivity of the Micralyne films changes by about four orders of magnitude in response to a similar change in surface illuminance. The plot shows that their dark conductivity is 10^−10^ cm^−1^ Ω^−1^. In general, a-Si:H films with dark conductivity from 10^−9^ to 10^−11^ cm^−1^ Ω^−1^ are of good quality [59]. A second y-axis gives the estimated current for a 10 × 10 *μ*m^2^ pixel, *i.e.*, for pixel dimensions more suitable for imaging than the ones actually used (110 × 110 *μ*m^2^), assuming 1V is applied across a 500 nm film. Currents in this range may be easily sensed by CMOS circuits. Figure 9(b) proves that the Micralyne a-Si:H films are suitable for imaging in the visible band with the readout done using conventional CMOS circuits.

There is one more factor to note. Steabler and Wronski [60] found that, when exposed to light, there is a gradual decrease in the photocurrent and dark current of a-Si:H films. This change can be reversed by annealing the films in a temperature that is slightly lower than their deposition temperature. Extensive research has been done on the Steabler-Wronski effect (SWE) by various groups around the world (see, for example, Stutzmann *et al.* [61]). We are not certain to what extent our VI-CMOS image sensor is affected by the SWE. However, our main purpose is prototype fabrication and proof of functionality. Different light-sensitive devices may be used in future.

### 3.3. Flip-Chip Bonding

Figure 3 shows finished CMOS and photodetector dies. UBM bond pads were fabricated on the photodetector dies, both on the a-Si:H surface, where they are arranged in a 20 × 24 array, and on the exposed ITO at the array periphery. Design and fabrication of these bond pads are discussed in a CMC application note [62]. The finished dies were sent to a flip-chip contractor, who deposited TSM on the interior bond pads of the CMOS dies, formed indium-based solder bumps on the UBM bond pads, and assembled several prototypes by flip-chip bonding.

Difficulties encountered by the contractor suggest that, for future projects of a similar nature, it is preferred that the UBM bond pads be prepared at the contractor’s facility. The process developed there for the UBM includes deposition of a titanium adhesion layer and a thick aluminum layer. This is followed by electroless-nickel immersion-gold plating. It is also preferred that undiced glass substrates with photodetector arrays are sent rather than diced photodetector dies. Some dies were damaged as they were too small to handle. After formation of the solder bumps, the flip-chip contractor can dice the substrates into dies at his facility.

## 4. Experimental Results

A PCB was designed to test the VI-CMOS image sensor prototype. For data conversion, the PCB includes a 16-bit commercial ADC (Texas Instruments ADS8411). Activation of the image sensor and the ADC is accomplished with an Altera Cyclone II FPGA board, which communicates with a PC through a QuickUSB board from Bitwise Systems. The FPGA is programmed to scan the array using the row and column address decoders. After a new address is placed, control signals are sent to the ADC to sample the analog output line of the image sensor. Data placed on the ADC output bus is read at video rate by the FPGA and sent to a PC.

**Figure 10.**
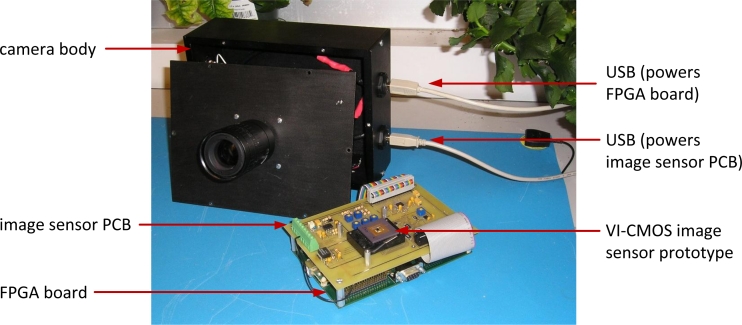
Digital camera that was designed and built to test the VI-CMOS prototype. Data transfer is done between the FPGA and a PC.

In the PC, an application has been developed in MATLAB and C++ to process the data in real time and display it on the screen. To capture scenes, the image sensor PCB is placed on the top of the FPGA board, and the two are accommodated in a camera body that was designed for this purpose. The two boards are powered by PC universal serial bus (USB) ports. A photo of the disassembled camera is shown in Figure 10.

**Figure 11.**
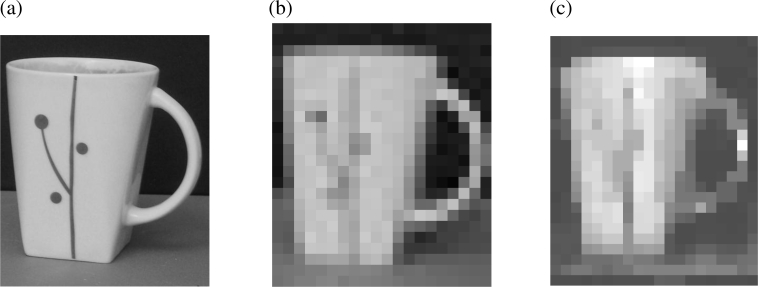
Example images: **(a)** taken with a commercial CCD camera; **(b)** same as previous but with the resolution changed to match that of the prototype; and **(c)** taken with the VI-CMOS prototype.

The main drawback of the VI-CMOS prototype is its low spatial resolution. To demonstrate the effect of working with large pixels, the same scene was photographed with a commercial CCD camera (an Olympus D-575 with 3.2 megapixels and a 1/2.5” sensor) and with the prototype. The original photo taken with the CCD camera is shown in Figure 11(a). Figure 11(b) shows the image obtained after the original photo has been processed to match the resolution of the VI-CMOS prototype, *i.e.*, an array of 20 × 24 pixels with 110 *μ*m pitch. A photo of the mug as taken with the prototype is shown in Figure 11(c).

Signal and noise properties of a digital camera define four important measures that affect the overall image quality: the signal-to-noise ratio (SNR), the signal-to-noise-and-distortion ratio (SNDR), the dynamic range (DR), and the dark limit (DL). Noise sources exist in the imaging system and in the scene. They can be divided into two types: temporal noise and fixed-pattern-noise (FPN). The SNR considers only the temporal noise, whereas the SNDR considers both temporal and fixed-pattern noise, which are assumed to be uncorrelated. The DL is the luminance level for which the SNDR is 0 dB. At this operating point, the signal and noise power are equal. The DR is the range of luminances that the imaging system can capture in a single frame with SNDR greater than 0 dB.

To characterize the signal and noise properties obtained with the VI-CMOS prototype, the camera was pointed at a uniformly illuminated scene. A light bulb with colour temperature of 2,700 K was used as the light source. The image plane illuminance was varied by changing the aperture diameter (or the f-stop number) of the pupil. Nine calibrated values are defined on the lens (Fujinon CF25HA-1) that is used with the camera. A neutral density filter (Hoya ND400) with attenuation ratio of 400 was used in combination with the pupil. The scene luminance captured by the camera was measured with a light meter (Sekonic L-758CINE) in cd/m^2^.

For these measurements, the image sensor was configured to connect the vertical photodetectors to the input nodes of the standard logarithmic-response circuits, and data was read through the output lines of those circuits. Twenty frames sampled at a frame rate of 70 Hz were read and recorded at each luminance level. The data was used for statistical calculations, *i.e.*, calculations of means and standard deviations, that are needed to determine the signal and noise properties. The average response of each pixel is used as calibration data for a real-time FPN-correction algorithm.

**Figure 12.**
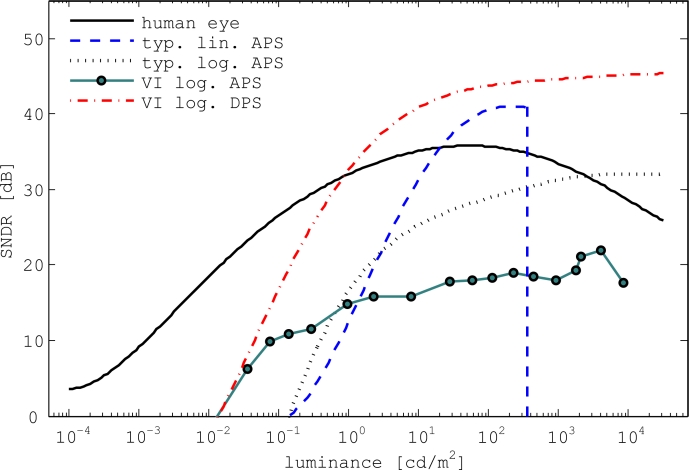
SNDR curves of the human eye, typical linear and logarithmic CMOS APS, and the VI-CMOS APS. The expected curve from a VI-CMOS DPS is also shown.

Figure 12 shows SNDR curves of the human eye and conventional CMOS APS cameras. It also shows the SNDR curve obtained with the VI-CMOS prototype. When enough time is given for adaptation, the DR of the human eye extends at least 170 dB. The peak SNDR of the human eye is 36 dB, which is reached in typical office luminance [63]. Human vision has three regions of operation [64]. Scotopic vision, or dark vision, occurs for luminances lower than 0.001 cd/m^2^, and photopic vision, or color vision, occurs for luminances higher than 3 cd/m^2^. For luminances between these thresholds, the human eye operates in a transition mode called mesopic vision. In this region, the response to colour gradually deteriorates as luminance decreases.

Cameras with a linear CMOS APS or a CCD sensor can achieve high SNDR but have a low DR, whereas a logarithmic CMOS APS is characterized by a high DR but low SNDR [65]. Assuming parameters of a conventional lens, data provided for the image plane illuminance at which the SNDR of an image sensor is 0 dB can be used to estimate the DL of a digital camera built with that sensor. Janesick [66] and Hoefflinger [67], for example, reported values obtained experimentally with linear and logarithmic CMOS APS cameras respectively. One may conclude from Figure 12 that the prototype has a better (lower) DL than a typical CMOS APS, and a better (higher) DR than a linear CMOS APS. However, its peak SNDR is low.

In electronic image sensors, conversion of analog signals generated by photodetectors into digital signals can be done at four different levels. At board level, one or more ADCs are placed on the PCB. At chip level, one or more ADCs are fabricated on the same chip as the sensor array. At column level, there are one or two ADCs at the edge of each column and, at pixel level, each pixel contains an ADC to make a digital pixel sensor (DPS). In general, the longer the path an analog signal needs to travel to reach an ADC, the greater the noise it accumulates. Increased noise translates into poorer performance in terms of SNR, SNDR, DR, and DL.

Although chip and column level data conversion are typically used in a commercial CMOS APS, data conversion was done at board level here with the VI-CMOS prototype. In a parallel project, Mahmoodi designed, built, and tested a logarithmic CMOS DPS, where each pixel includes a delta-sigma ADC [68]. Characterization of this image sensor shows that its DL is comparable to that of conventional CMOS APS, and its DR is comparable to that of logarithmic CMOS APS. However, thanks to the pixel-level data conversion, the SNDR is significantly improved when compared to typical logarithmic CMOS APS. Mahmoodi reports a peak SNDR of 46 dB. Therefore, Figure 12 also shows the expected SNDR curve from a VI-CMOS image sensor that has a photodetector optimized for low DL, a logarithmic response that achieves high DR, and pixel-level data conversion for high SNDR.

## 5. Conclusion

Image sensors include photodetectors and mixed-signal circuits, which involve devices with different requirements. Vertical integration of these devices means each tier may be fabricated in a different process. This enables advanced circuits in each pixel without sacrificing spatial resolution. Advanced pixel-level circuitry is essential for improving the overall performance of image sensors. This paper focuses on VI-CMOS image sensors made by flip-chip bonding; they are composed of a CMOS die and a photodetector die. Other fabrication methods for VI-CMOS image sensors are possible.

The main difference between a CMOS die of a VI-CMOS image sensor and a CMOS image sensor is that, with the former, each pixel needs a bond pad for a vertical photodetector and does not need a lateral photodetector. It is desirable to leave the light-sensitive semiconductor unpatterned in the photodetector die of a VI-CMOS image sensor. This results in a preference for feedback active pixels in the CMOS die, whereby potential differences between adjacent photodetector contacts are attenuated to reduce pixel crosstalk.

The design of photodetectors for VI-CMOS image sensors, especially those fabricated by flip-chip bonding, has many more degrees of freedom than the design of photodetectors for CMOS image sensors. Choices need to be made regarding materials used for the handle substrate, the transparent electrode, and the light-sensitive devices. One must also choose the light-sensitive device type, which may be a photoconductor, photodiode, or phototransistor. With all this freedom, photodetectors may be optimized for various applications.

In addition to general design and fabrication principles, supported by extensive references, this work presents a specific VI-CMOS image sensor prototype. To make the prototype, a CMOS die was designed for a commercial process, and a photodetector die was designed for a custom process. The CMOS die was fabricated by Dalsa through CMC Microsystems, and the photodetector die was fabricated at the University of Alberta Nanofab and Micralyne Inc. Finally, the two dies were assembled by a flip-chip contractor through CMC.

The VI-CMOS prototype includes two sets of CMOS circuits in each pixel. The first is a feedback logarithmic-response circuit, and the second is a standard logarithmic-response circuit. Each pixel also includes both a vertical MSM photodetector, which uses an unpatterned a-Si:H film, and a lateral CMOS photodiode. Optoelectronic properties of the Micralyne a-Si:H films were reported. The films proved excellent for visible-band imaging.

An imaging system has been developed to test the prototype. It is based on a QuickUSB Altera FPGA board that communicates with a PC in real-time. Characterization results of the signal and noise properties at video rates show that the prototype has a lower dark limit and a higher dynamic range than a conventional CMOS APS. The SNDR, however, is low. While data conversion with the VI-CMOS prototype is done at board level, a logarithmic CMOS DPS has recently shown an SNDR greater than 40 dB. Therefore, a logarithmic VI-CMOS DPS would have superior signal and noise properties.

The main drawback with the prototype is a low spatial resolution due to large pixels. Even if fine-pitch flip-chip bonding cannot be accessed by Canadian researchers in the near future, there are applications where large pixels are acceptable. For example, in medical X-ray imaging, which is a lens-less imaging technique, pixel pitches are of several tens of microns. Optimization of the photodetectors for a lower dark limit means that patients would be exposed to a lower X-ray dosage. Another advantage of the presented approach is its robustness. As long as contact dimensions and electrical interfaces are preserved, the same CMOS die may be bonded to various sensor dies, which are not limited to photodetector dies.

## Figures and Tables

**Table 1.** Coefficients of thermal expansion (CTEs) of silicon and substrates that are transparent in the visible band [22].

Substrate material	CTE [10^−6^ K^−1^]		
	@ 200 K	@ 293 K	@ 500 K
Silicon	1.5	2.6	3.5
Glass, borosilicate	2.7	2.8	3.3
Glass, fused-silica	0.1	0.5	0.6
Glass, soda-lime	-	7.5	-
Quartz, single crystal, ‖ c-axis	5.2	6.8	11.4
Quartz, single crystal, ⊥ c-axis	10.3	12.2	19.5
Sapphire, single crystal, ‖ c-axis	4.1	4.8	7.9
Sapphire, single crystal, ⊥ c-axis	6.6	7.4	8.3

**Table 2.** Optoelectronic properties of the three TCOs that are most commonly used [26].

Material	Band Gap [eV]	Conductivity [Ω^−1^cm^−1^]	Carrier Concentration [cm^−3^]	Mobility [cm^2^V^−1^s^−1^]
In_2_O_3_	3.75	10^4^	> 10^21^	35
ZnO	3.35	8 × 10^3^	> 10^21^	20
SnO_2_	3.60	5 × 10^3^	> 10^20^	15

**Table 3.** Abbreviations and full names of polymers commonly used in organic electronic devices.

Abbreviation	Full Name
CuPc	copper(II) phthalocyanine
PC60BM	(6,6)-phenyl-C61-butyric acid methyl ester
PDDTT	poly(5,7-bis(4-decanyl-2-thienyl)-thieno (3,4-b)diathiazole-thiophene-2,5)
PEDOT	poly(3,4-ethylenedioxythiophene)
PSS	poly(styrenesulfonate)
P3HT	poly(3-hexylthiophene)
